# The universal accumulation of *p*-aminophenol during the microbial degradation of analgesic and antipyretic acetaminophen in WWTPs: a novel metagenomic perspective

**DOI:** 10.1186/s40168-025-02065-2

**Published:** 2025-03-07

**Authors:** Chao-Fan Yin, Piaopiao Pan, Tao Li, Xin Song, Ying Xu, Ning-Yi Zhou

**Affiliations:** 1 State Key Laboratory of Microbial Metabolism, Joint International Research Laboratory of Metabolic & Developmental Sciences, and School of Life Sciences & Biotechnology, Shanghai Jiao Tong University, Shanghai, 200240 China; 2 State Key Laboratory of Soil and Sustainable Agriculture, Institute of Soil Science, Chinese Academy of Sciences, Nanjing, 211135 China

**Keywords:** Acetaminophen, Amidases, Environmental fate, Metagenome, Microbiota-pollutant interactions, WWTPs

## Abstract

**Background:**

Acetaminophen, a widely used analgesic and antipyretic drug, has become a significant aquatic micro-pollutant due to its extensive global production and increased consumption, particularly during the COVID-19 pandemic. Its high-water solubility leads to its pervasive presence in wastewater treatment plants (WWTPs), posing substantial risks to the environment and human health. Biological treatment is one of the promising approaches to remove such pollutants. Although previous studies have isolated acetaminophen-degrading pure cultures and proposed catabolic pathways, the interactions between microbiotas and acetaminophen, the distribution feature of acetaminophen degradation genes, and the gene-driven fate of acetaminophen in the real-world environment remain largely unexplored.

**Results:**

Among the water samples from 20 WWTPs across China, acetaminophen was detected from 19 samples at concentrations ranging from 0.06 to 29.20 nM. However, *p*-aminophenol, a more toxic metabolite, was detected in all samples at significantly higher concentrations (23.93 to 108.68 nM), indicating the presence of a catabolic bottleneck in WWTPs. Metagenomic analysis from both the above 20 samples and global datasets revealed a consistently higher abundance of initial acetaminophen amidases compared to downstream enzymes, potentially having explained the reason for the bottleneck. Meanwhile, a close correlation between initial amidases and Actinomycetota revealed by genome-based taxonomy suggests a species-dependent degradation pattern. Additionally, a distinct amidase ApaA was characterized by newly isolated *Rhodococcus* sp. NyZ502 (Actinomycetota), represents a predominant category of amidase in WWTPs. Significant phylogenetic and structural diversity observed among putative amidases suggest versatile acetaminophen hydrolysis potential in WWTPs.

**Conclusions:**

This study enhances our understanding of acetaminophen’s environmental fate and highlights the possible occurrence of ecological risks driven by imbalanced genes in the process of acetaminophen degradation in global WWTPs.

Video Abstract

**Supplementary Information:**

The online version contains supplementary material available at 10.1186/s40168-025-02065-2.

## Background

Acetaminophen (*N*-acetyl-*p*-aminophenol, APAP), commonly known as paracetamol, is one of the most widely used analgesic and antipyretic drugs. With an annual global production exceeding 160,000 tons, China alone contributes more than 50% [1]. The COVID-19 pandemic has notably escalated APAP production and consumption [2], with domestic daily production peaking at 190 million tablets. Its high water solubility facilitates its accumulation in aquatic environments via direct disposal of excess medications, human and animal feces, and discharge from hospitals and pharmaceutical manufacturing [3].

APAP has been frequently detected in wastewater, surface water, groundwater, and even drinking water, with concentrations ranging from ng/L to mg/L [1], categorized as a significant class of aquatic micro-pollutants. Environmental exposure to APAP poses substantial risks to aquatic organisms at detectable concentrations (mg/L) and can potentially impact human health through bioaccumulation and transmission through the food chain [4]. Its transformation products, such as *N*-acetyl-*p*-benzoquinone imine (NAPQI), are hepatotoxic, contributing to liver injury upon excessive exposure [5]. Prenatal exposure to APAP has also been linked to adverse fetal development and increased risks of neurodevelopmental and genitourinary disorders [6].

Microorganisms play a crucial role in the environmental element cycling of human-related compounds including manmade pharmaceuticals such as APAP. Previous studies have primarily focused on isolating APAP-degrading strains, which turned out to be predominantly Gram-negative bacteria, and proposing their catabolic pathways [7, 8]. It is generally accepted that the APAP degradation pathway was initiated with the hydrolysis of APAP by a group of amidases, leading to the formation of a common intermediate *p*-aminophenol (PAP). This intermediate can be further metabolized through multiple routes, such as conversion to hydroquinone (HQ) or 1,2,4-benzenetriol (BT), which are subsequently metabolized via common pathways converging on the tricarboxylic acid cycle. Although these studies have provided valuable insights into the catabolic mechanisms of APAP at lab scales, they often overlooked the complex interactions between functional microbial genera/associated genes and the fate of APAP in real-world environments. Particularly in aquatic environments with the prevalence of APAP, the presence and contribution of functional genera, along with their gene compositions, remain underexplored. The distribution patterns of these functional genes, their correlation with the fate of acetaminophen, and the underlying mechanisms of environmental transformation mediated by these genes have yet to be fully elucidated.

Meantime, enzymes involved in the degradation of APAP have been shown to exhibit considerable diversity and broad substrate specificity. For instance, two identical amidases (AAA [9] and Ppa [10]) were shown to catalyze the hydrolysis of a variety of aryl amide compounds, including APAP [9, 10] and swep [10]. Additionally, amidases responsible for the degradation of pesticides or herbicides, including AmpA [11], DmhA [12], Mah [13], as well as the triclocarban (TCC)-degrading enzyme TccA [14], have also been demonstrated active against APAP. Besides, two additional amidases ApaH1 and ApaH2 involved in a novel APAP degradation pathway were characterized very recently [15]. These findings highlight the diverse repertoire of APAP-degrading amidases and also underscore the potential for functional gene mining to identify additional enzymes capable of degrading APAP in the environment.

It is generally accepted that the diverse metabolic potentials of the environmental microorganisms are genera- and genes-dependent [16], which is crucial for understanding and evaluating the environmental fate of APAP in this case. For this purpose, we here collected water samples from as many as 20 wastewater treatment plants (WWTPs) across China to quantify APAP and its metabolites. Central to our research is the application of metagenomic analysis for a comprehensive examination of the microbial communities and functional genes involved in APAP degradation. Functional microbial isolation, enzyme assays, and microbial diversity analysis were also employed to further elucidate the mechanisms driving APAP transformation in WWTP environments. This study not only enhances our understanding of the environmental fate of APAP but also lays the groundwork for developing sustainable technologies for the removal of APAP as well as other pharmaceutical micro-pollutants.

## Methods

### Reagents

Acetaminophen (APAP) and *p*-aminophenol (PAP) were purchased from Beijing InnoChem Science & Technology Co., Ltd. (Beijing, China). Acetanilide was purchased from Shanghai Aladdin Biochemical Technology Co., Ltd. (Shanghai, China). 4-chloroacetanilide, 4-nitroacetanilide, and *N*-(3,4-dichlorophenyl) propanamide (commonly known as propanil) were purchased from Bide Pharmatech Co., Ltd. (Shanghai, China). *Escherichia coli* (*E.Ccoli*) was cultured aerobically in lysogeny broth (LB) [17]. APAP-degrading strains were isolated and cultured in mineral salts medium (MM) [18], with APAP as the sole carbon source at 30 °C. Both MM and LB agar media were prepared by adding 1.5% (w/v) agar.

Sampling and analysis.

Wastewater samples were collected from wastewater treatment plants (WWTPs) in 20 cities across China, as detailed in Additional file 1: Table S1. For the quantitative analysis of APAP and PAP in these samples, the wastewater was first filtered using 0.22 µm filters. The filtered samples were then subjected to Liquid Chromatography-Mass Spectrometry (LC–MS) analysis as described below.

### LC–MS analysis

Wastewater samples were analyzed using an Agilent 1260 Liquid Chromatography (LC) system coupled with an Agilent 6470 Triple Quadrupole (QqQ) mass spectrometer (Agilent Technologies, Santa Clara, CA, USA).

Chromatographic separation was performed on an Agilent ZORBAX SB-C18 column (250 × 4.6 mm, 5 µm, Agilent Technologies), maintained at 25 °C. A gradient elution was applied at a constant flow rate of 0.4 mL/min, using mobile phase A (0.1% (v/v) formic acid in deionized water) and mobile phase B (acetonitrile). The gradient program was as follows: 0–14 min, 20% B; 14–17 min, 20–100% B; 17–17.5 min, 100–20% B; 17.5–21 min, 20% B. The total run time for the chromatographic analysis was 21 min, with an injection volume of 10 µL.

The mass spectrometer was operated in positive electrospray ionization (ESI) mode, utilizing Agilent Jet Stream Technology (AJS ESI) and multiple reaction monitoring (MRM) mode. The ion source parameters were set as follows: capillary voltage at 4.0 kV, source temperature at 300 °C, drying gas flow rate at 5 L/min, nebulizer pressure at 45 psi, sheath gas temperature at 250 °C, and sheath gas flow rate at 11 L/min.

### DNA extraction, library construction, and metagenomic sequencing

Total genomic DNA was extracted from wastewater samples using the Mag-Bind® DNA kit (Omega Bio-tek, Norcross, GA, USA) following the manufacturer’s instructions. The concentration and purity of extracted DNA were assessed using a TBS-380 fluorometer and a NanoDrop 2000 spectrophotometer. The quality of the DNA extract was verified on a 1% agarose gel.

The DNA was fragmented to an average size of approximately 400 bp using a Covaris M220 ultrasonicator (Gene Company Limited, China) for paired-end library construction. The paired-end library was constructed using the NEXTFLEX Rapid DNA-Seq Kit (Bioo Scientific, Austin, TX, USA), with adapters containing the full complement of sequencing primer hybridization sites ligated to the blunt ends of the DNA fragments.

Paired-end sequencing was performed on an Illumina NovaSeq platform (Illumina Inc., San Diego, CA, USA) at Majorbio Bio-Pharm Technology Co., Ltd. (Shanghai, China), using the NovaSeq 6000 S4 Reagent Kit v1.5 (300 cycles) according to the manufacturer’s instructions (www.illumina.com). Sequence data associated with this project have been deposited in the NCBI Sequence Read Archive (SRA) under BioProject accession number: PRJNA1148826.

### Metagenomic quality control and assembly

Paired-end Illumina reads were processed to remove adapters and low-quality sequences using fastp (v0.23.4) [19]. The cleaned reads were then assembled into contigs using MEGAHIT (v1.2.9) [20]. Contigs shorter than 500 bp were discarded, and the remaining contigs were retained for subsequent gene prediction and annotation.

### Taxonomic profiling and gene annotation

To generate taxonomic profiles of microbial communities from different wastewater samples, clean reads were annotated using Kraken2 (v2.1.3) [21], with abundance estimates obtained through Bracken (v2.9). Assembled contigs were used for gene prediction via Prodigal (v2.6.3) [22] with the “-c -p meta” option. Homologs of target genes in each sample were identified using Diamond BLASTP [23], applying thresholds of > 30% sequence identity and > 70% coverage. Gene abundance was quantified using CoverM, with the bwa-mem2 algorithm for reads mapping. Taxonomic classification of each protein sequence was determined through Diamond BLASTP against the NCBI nr database.

### Metagenomic binning, GTDB classification, and functional annotation

Assembled contigs from each sample were individually binned using MaxBin2 [24] (v2.2.6) and Metabat2 (v2.12.1) [25]. The resulting bins were combined and dereplicated using dRep (v3.5.0) [26]. Bin quality was assessed with CheckM (v1.2.2) [27], retaining a total of 211 high-quality metagenome-assembled genomes (MAGs) with genome completeness > 90% and contamination < 5% for further analysis. The abundance of each MAG was quantified as genome copies per million reads (GPM) using the Quant_bins module in MetaWRAP [28] with Salmon (v0.13.1). Protein-coding genes within each MAG were predicted using Prodigal (v2.6.3) [22], and potential acetaminophen catabolic genes were identified with Diamond (v2.1.8) [23], applying a criterion of > 30% sequence identity and > 70% coverage. Taxonomic classification of all MAGs was performed using the classify_wf module in GTDB-Tk (v2.2.4) [29].

### Isolation and characterization of APAP-degrading bacterial strains

A water-sludge mixture from sewage treatment plants was introduced into a mineral salts medium (MM) containing 2 mM APAP to serve as an enrichment medium. The mixture was incubated at 30 °C with shaking at 180 rpm. A portion of the turbid culture was subcultured into fresh MM with 2 mM APAP for a second round of enrichment. This process was repeated for a total of three enrichment rounds. After the final enrichment, the culture was spread onto LB agar plates. Isolated colonies with distinct morphologies were selected and subjected to degradation phenotype characterization. Taxonomic classification of the isolates was performed based on 16S rRNA gene sequencing, using universal primers 27F and 1492R, as detailed in Additional file 1: Table S3.

### Growth and degradation of APAP by isolated degraders

The growth of APAP-degrading strains in mineral salts medium (MM) supplemented with 1 mM APAP was monitored through colony counting as previously described [30]. At various intervals, cultures were collected and analyzed using high-performance liquid chromatography (HPLC) under the conditions outlined above. To identify APAP and its intermediate products, an Agilent 6230 time-of-flight mass spectrometer (TOF–MS, Agilent Technologies, Santa Clara, CA, USA) was employed. The mass spectrometer operated in positive electrospray ionization (ESI) mode. Intermediate products were verified by comparing their mass spectra to those of authentic standards.

### Genome sequencing

Genomic DNA from *Rhodococcus* sp. strain NyZ502 was extracted using the Bacterial DNA Extraction Kit (magnetic beads) (Majorbio, Shanghai, China). Sequencing was performed using a combination of PacBio Sequel II and Illumina platforms to obtain high-quality genomic data. The sequencing data were analyzed using the Majorbio Cloud Platform (http://cloud.majorbio.com). The genome sequence of strain NyZ502 has been deposited in the NCBI database under the BioProject accession number PRJNA1147891.

### Gene cloning, protein expression and purification

Amidase genes for overexpression were cloned using a One Step Cloning Kit (Vazyme Biotech Co., Ltd.). The genes *aaa* [9, 31] and *ppa* [10] (GenBank accession number FJ755834.2 and MH822147.1, respectively), which share 100% nucleotide identity and originate from different bacterial strains, were also found in *Pandoraea* sp. strain NyZ501, which was referred to as *aaa* for normalization. The studied amidase genes were amplified and inserted into the pET-28a ( +) or pTIPQC1vectors as necessary. The resultant plasmids pET-*aaa* and pTIP-*apaA* were transformed into *Escherichia coli* BL21 (DE3) and *Rhodococcus erythropolis* L88 using standard heat shock transformation and electroporation procedures [32], respectively.

For protein expression and purification in *E. coli*, the strain was grown in an LB medium supplemented with 50 µg/mL kanamycin at 37 °C and 180 rpm. When the culture reached an OD600 of 0.6, protein expression was induced with 0.1 mM isopropyl-β-D-thiogalactopyranoside, followed by incubation at 16 °C overnight (16 h) at 150 rpm.

For protein expression in *R. erythropolis* L88, the strain was grown in LB medium supplemented with 25 µg/mL chloramphenicol at 30 °C and 200 rpm. Upon reaching an OD600 of 0.6, protein expression was induced with 1 µg/mL thiostrepton, followed by incubation at 30 °C and 200 rpm for 24 h.

The induced cells were washed and resuspended in phosphate buffer (PB; 100 mM Na_2_HPO_4_/NaH_2_PO_4_, pH 7.4; 200 mM NaCl; and 10% [v/v] glycerol) and then subjected to sonication. The lysate was centrifuged at 13,000 × *g* for 40 min at 4 °C, followed by filtration through 0.45 µm filter membranes to obtain the cell extracts. His_6_-tagged protein purification was performed using Ni–NTA gravity chromatography columns (BBI, Sangon Biotech Co., Ltd.). The columns were sequentially washed with 25 mM, 50 mM, and 80 mM imidazole, and the recombinant His₆-tagged proteins were eluted with 250 mM imidazole dissolved in PB. Imidazole was subsequently removed from the protein solution by ultrafiltration. The recombinant His₆-tagged proteins were then separated using sodium dodecyl sulfate–polyacrylamide gel electrophoresis (SDS-PAGE) and quantified using a Nano-300 spectrophotometer (Allsheng Instruments Co., Ltd., Hangzhou, China) at A₂₈₀ nm.

### Enzyme assay

The enzyme assay was conducted using a Lambda 25 spectrophotometer (PerkinElmer/Cetus, Norwalk, CT). The reaction mixtures (0.5 mL) contained 0.5 μg purified amidases and 0.2 mM APAP in 100 mM phosphate buffer (pH 7.4). The assay was initiated by the addition of substrates.

For kinetic assays of amidases, specific activities towards APAP and other amide-containing compounds were measured at varying substrate concentrations. Data from three independent experiments were fitted to the Michaelis–Menten equation using OriginPro 8 software (OriginLab, MA, USA). One unit of enzyme activity was defined as the amount of enzyme required to catalyze the consumption of 1 μmol of substrate per minute at 30 °C. Specific activities are expressed as units per micromole of protein.

The enzyme assay was performed at different pH levels (3.5–10.5) and temperatures (4–55 °C) to determine the optimal reaction conditions. The thermal stability of the amidase was evaluated by pre-incubating the enzyme at temperatures ranging from 4 to 55 °C for 30 min, followed by the standard enzyme assay at the optimal temperature.

### Phylogenetic analysis and sequence similarity network

Homologous sequences of putative APAP amidases were identified by performing a BLASTP search against metagenomic data from WWTP samples using known functional APAP amidases as query sequences. Homologs with over 30% sequence identity and at least 70% coverage were selected. These sequences were aligned using Clustal Omega [33]. The aligned sequences were then used to construct a phylogenetic tree with FastTree [34], which was subsequently visualized and annotated using tvBOT [35].

The sequence similarity network (SSN) was generated using the Enzyme Function Initiative Enzyme Similarity Tool (EFI-EST) [36]. Sequences underwent an all-by-all BLAST, and the SSN was constructed with an alignment score threshold of 70. The SSN was visualized using Cytoscape [37]. The structures of the amidases were predicted using AlphaFold3 [38].

### Retrieval and analysis of global WWTP metagenomes

A total of 117 metagenomic samples from global WWTPs were retrieved from the NCBI Sequence Read Archive (SRA). The geographic coordinates (latitude and longitude) of each sample were obtained using the Efetch tool. Raw sequencing reads of each sample were subjected to quality control and assembly, as described above. The analysis focused on enzymes from APAP-utilizing microorganisms involved in the degradation of APAP. These included the initial APAP amidase (AAA [9], ApaH1, ApaH2 [15], and ApaA) as well as secondary enzymes (ApdB [15], GuaD [39] and SadBC [40]) involved in PAP conversion. Using these enzymes as queries, all WWTP metagenomes were searched with the BLASTP algorithm, applying a threshold of sequence identity > 30% and query coverage > 70%. The abundance of each enzyme in the metagenomes was then quantified as transcripts per million (TPM) using CoverM.

### Statistical analysis

Statistical analyses were performed using SPSS. Data were analyzed using one-way ANOVA or *t*-test, as appropriate. Results are presented as mean ± standard deviation, and a *p*-value of less than 0.05 was considered statistically significant.

## Results

### APAP degradation and accumulation of its intermediate PAP in WWTPs

**Fig. 1:**
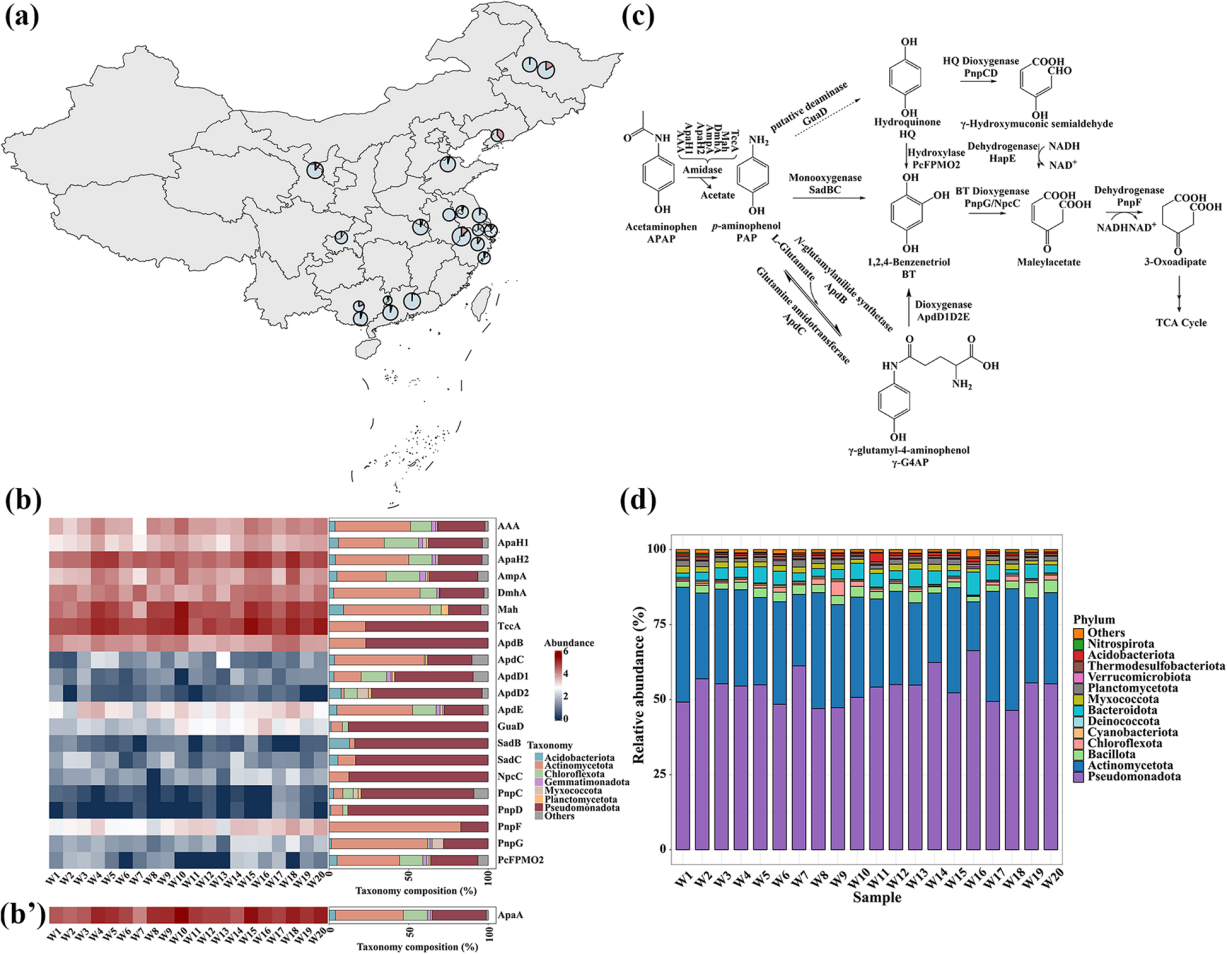
Distribution profiles of acetaminophen (APAP) and its intermediate *p*-aminophenol (PAP), degradation enzymes as well as their associated microbial communities. **a** Sampling locations and chemical profiles. The sampling locations for wastewater treatment plant (WWTP) samples are mapped using QGIS (https://qgis.org/). Pie charts show the relative concentrations of APAP (red) and its degradation product, PAP (blue), at each site (data available in Additional file 1: Table S1 with statistics analysis). Both APAP and PAP were detected at all sites except sample W9 where no APAP was detected, with PAP consistently present at higher concentrations than APAP. **b**, **b**’ Abundance of APAP degradation enzymes and their taxonomic composition. The left part displays the abundance of enzymes involved in APAP degradation, where each row corresponds to an enzyme and each column to a sample. Abundances were log-transformed (ln(tpm + 1)) and are represented using a blue-to-red gradient scale. The right part shows the taxonomic composition of each enzyme, annotated by BLAST, at the phylum level. **c** Proposed APAP degradation pathway. Initial degradation is catalyzed by amidases, including AAA [9], ApaH1, ApaH2 [15], AmpA [11], DmhA [12], Mah [13], and TccA [14]. Downstream steps are catalyzed by enzymes including GuaD (putative) [39], ApdB, ApdC, ApdD1D2E [15], SadBC [40], PcFPMO2 [45], PnpCD [46], PnpG [46], NpcC [47], HapE [48], and PnpF [46], as shown. **d** Taxonomic composition of WWTP samples. The stacked bar chart represents the taxonomic composition at the phylum level for each WWTP sample

It is known that *p*-aminophenol (PAP) is mainly formed as a hydrolytic product of APAP degradation [41, 42]. In WWTP samples detected here, PAP was found at significantly higher concentrations than its parent compound APAP. Specifically, the amounts of APAP and PAP together with their ratio in 20 wastewater samples across China are shown in Fig. 1a (data detailed in Additional file 1: Table S1 and Additional file 1: Fig. S1). APAP was detected in 19 samples, with concentrations varying from 0.06 to 29.20 nM. PAP, the major hydrolytic product of APAP, was present in all samples at higher concentrations, ranging from 23.93 to 108.68 nM. This indicates that the hydrolysis of APAP to PAP is significantly more rapid compared to the downstream degradation steps, possibly due to the higher abundance of APAP hydrolase and/or its higher enzymatic activity in WWTPs.

### Uneven richness of APAP degradation enzymes in WWTPs

The APAP degradation pathway, proposed based on previous studies [3, 43], begins with the initial hydrolysis of APAP by a diverse group of amidases, as shown in Fig. 1c. These amidases catalyze the conversion of APAP to PAP, which is further degraded through several routes as proposed in Fig. 1c. PAP can be converted to hydroquinone (HQ) by the putative deaminase GuaD [39], or to 1,2,4-benzenetriol (BT) via the two-component monooxygenase SadBC [40], or to γ-glutamyl-4-aminophenol (γ-G4A) via an *N*-glutamylanilide synthetase [15]. Subsequently, the pathway converges on the tricarboxylic acid cycle, with both HQ and BT serving as intermediates that enter common metabolic routes. To test whether the imbalance between APAP and PAP concentrations in WWTPs is linked to the distribution pattern of functional degradation genes, their encoded enzymes were searched against the metagenome of the WWTP samples using BLASTP (Fig. 1b). The results revealed that the amidases involved in the initial hydrolyzation during the APAP degradation were much more frequently found in WWTPs, whereas most downstream enzymes were rarely observed except ApdB. This suggests that the imbalanced distribution pattern of APAP degradation enzymes likely contributes to the accumulation of PAP in WWTPs.

PAP is considered more toxic to both the environment [42] and human health [44] than its parent compound APAP. Upon entering WWTPs, APAP is often converted to PAP through the action of amidases. Taxonomic analysis of WWTP metagenomes revealed distinct patterns between microbial phyla containing initial amidases and those with downstream enzymes (Fig. 1b). Among the bacteria-harboring initial amidases, the phylum Actinomycetota was significantly more abundant than any other phylum, suggesting a strong correlation between this phylum and the presence of these amidases. Notably, Actinomycetota was the second most abundant phylum (Pseudomonadota was the most) across all WWTPs tested (Fig. 1d), indicating a similar degradation behavior across different WWTPs. This aligns with the uneven presence patterns of APAP and its degradation intermediate PAP in these systems.

### Broad diversity of APAP amidases in WWTPs

**Fig. 2:**
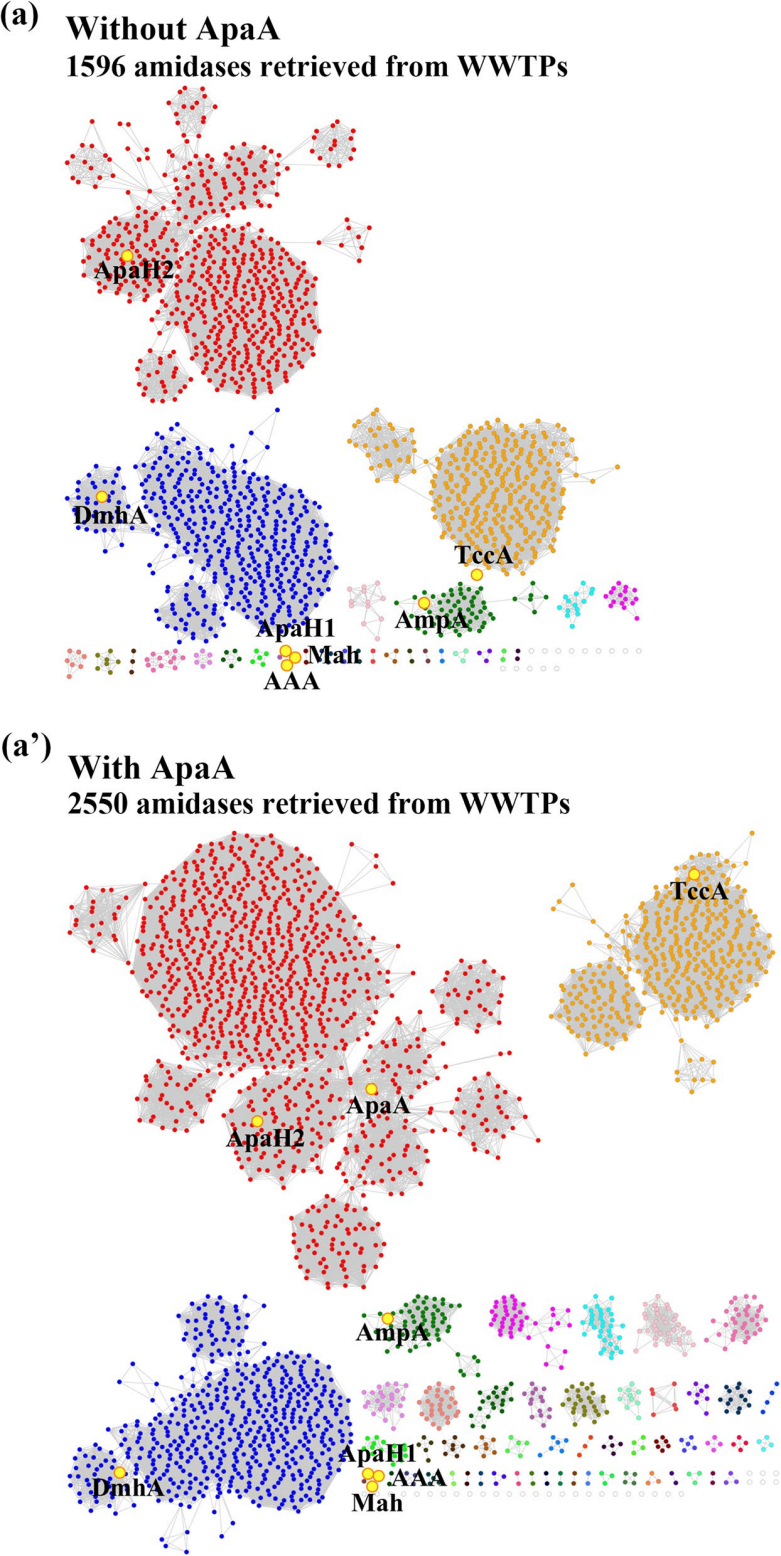
Sequence similarity network (SSN) of putative APAP amidases from WWTPs. The SSN was constructed using sequences retrieved by using previously reported APAP amidases as the query sequences (**a**) and using both previously reported amidases and ApaA characterized in this study as query sequences (**a’**). A total of 1596 and 2550 homologs from **a** and **a’** were retrieved respectively. Edges represent pairwise BLAST similarity with *E*-value < 10^−10^. The SSN clusters indicate that ApaA-alike sequences have significantly high richness in WWTPs

A previous study preliminarily proposed diverse APAP amidases present in a simulated system containing hospital-sourced sludge [43]. To further investigate whether a similar or even greater diversity of APAP amidases exists in actual WWTPs, all reported functional APAP amidases were used as query sequences to retrieve their homologs from the metagenome of WWTP samples, with thresholds of 30% amino acid sequence identity and 70% coverage. A total of 1596 sequences were retrieved, and a sequence similarity network (SSN) was constructed with an alignment score of 70 (Fig. 2a). Among the retrieved sequences, ApaH2 clustered with the most abundant amidase sequences in WWTPs, while DmhA, TccA, and AmpA clustered with the second, third, and fourth most abundant sequences, respectively. In contrast, ApaH1, AAA, and Mah together formed a distinct cluster, separated from other sequences. These results strongly suggest the diversity of unique groups and richness of uncharacterized APAP amidases in WWTPs, indicating the necessity of isolation of more APAP utilizers from WWTPs for discovering novel APAP amidases.

### APAP degradation by isolated bacterial strains from wastewater

Our recent interest in the microbial degradation mechanisms of pharmaceutical pollution has led to the successful isolation of a degrader of antidiabetic drug metformin from WWTPs [49]. Prior to the geochemical and metagenomic analyses of WWTP samples as aforementioned, two APAP utilizers, designated *Pandoraea* sp. strain NyZ501 and *Rhodococcus* sp. strain NyZ502, were isolated from an APAP-contaminated wastewater sample (Additional file 1: Fig. S2). Subsequently, an amidase gene was identified from strain NyZ501, which is identical to previously reported APAP-degrading genes *aaa* from a soil *Pseudomonas* strain (GenBank accession number: FJ755834.2) [9] and *Comamonas* sp. strain SWP-3 (MH822147.1) [10], revealed by PCR (Additional file 1: Fig. S3) and then sequencing validation. Interestingly, the genera *Pandoraea*, *Pseudomonas*, and *Comamonas* all belong to the phylum Pseudomonadota, which was identified as the most abundant phylum in aforementioned WWTP samples and also other wastewater systems [50]. This finding strongly suggests that this specific amidase gene (*aaa*) has been horizontally transferred among various Gram-negative bacterial strains, particularly within the Pseudomonadota phylum, likely as an adaptive response to amido bond-containing pharmaceutical pollutants like APAP [51].

**Fig. 3:**
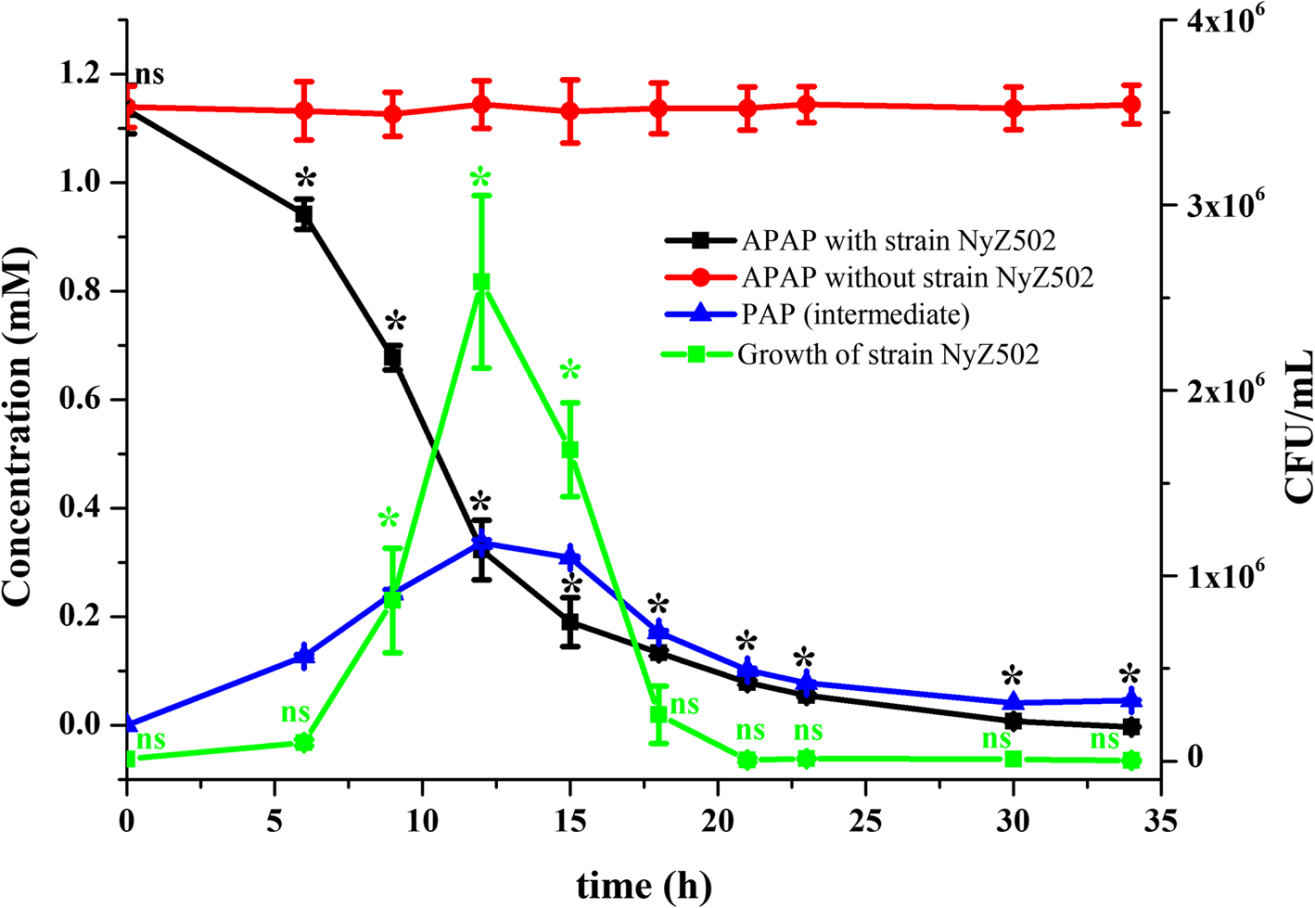
Characterization of APAP degradation by *Rhodococcus* sp. strain NyZ502 and identification of a degradation intermediate. Consumption of acetaminophen and cell growth, along with the temporal accumulation of the degradation intermediate PAP. APAP without inoculations remains unchanged over time. The green * indicates a significantly higher CFU count at this time point compared to others (*p* < 0.05), while green ns indicates no significant difference. The black * indicates a significantly decreased APAP concentration at this time point compared to the control group, and the black ns indicates no significant difference in APAP concentration at this time point

In contrast, no *aaa* gene was detected by PCR in the other new isolate *Rhodococcus* sp. strain NyZ502 (Additional file 1: Fig. S3), which belongs to the second most abundant phylum Actinomycetota (Gram-positive) in WWTPs. This suggests the probable presence of a distinct functional APAP amidase within this strain belonging to phylum Actinomycetota, making it a prime candidate for further investigation into its degradation phenotype and associated degradation genes in particular. As demonstrated in Fig. 3, when strain NyZ502 was cultured in minimal medium (MM) with APAP (final concentration of 1 mM), APAP was evidently biotransformed over time, with PAP detected as an intermediate product via LC–MS (Additional file 1: Fig. S4). Interestingly, the biomass of strain NyZ502 initially increased but then decreased as APAP was depleted, probably because that the strain either harbors incomplete gene set for the entire APAP degradation or PAP derivatives restrict its growth [8]. The former was subsequently evidenced by its inability to grow on PAP (data not shown). The reduction of PAP after 12 h may be caused by spontaneous oxidation due to its instability, especially at concentrations greater than 50 mg/L (approximately 0.45 mM), as previously reported [41].

### Characterization of APAP amidases ApaA

**Fig. 4:**
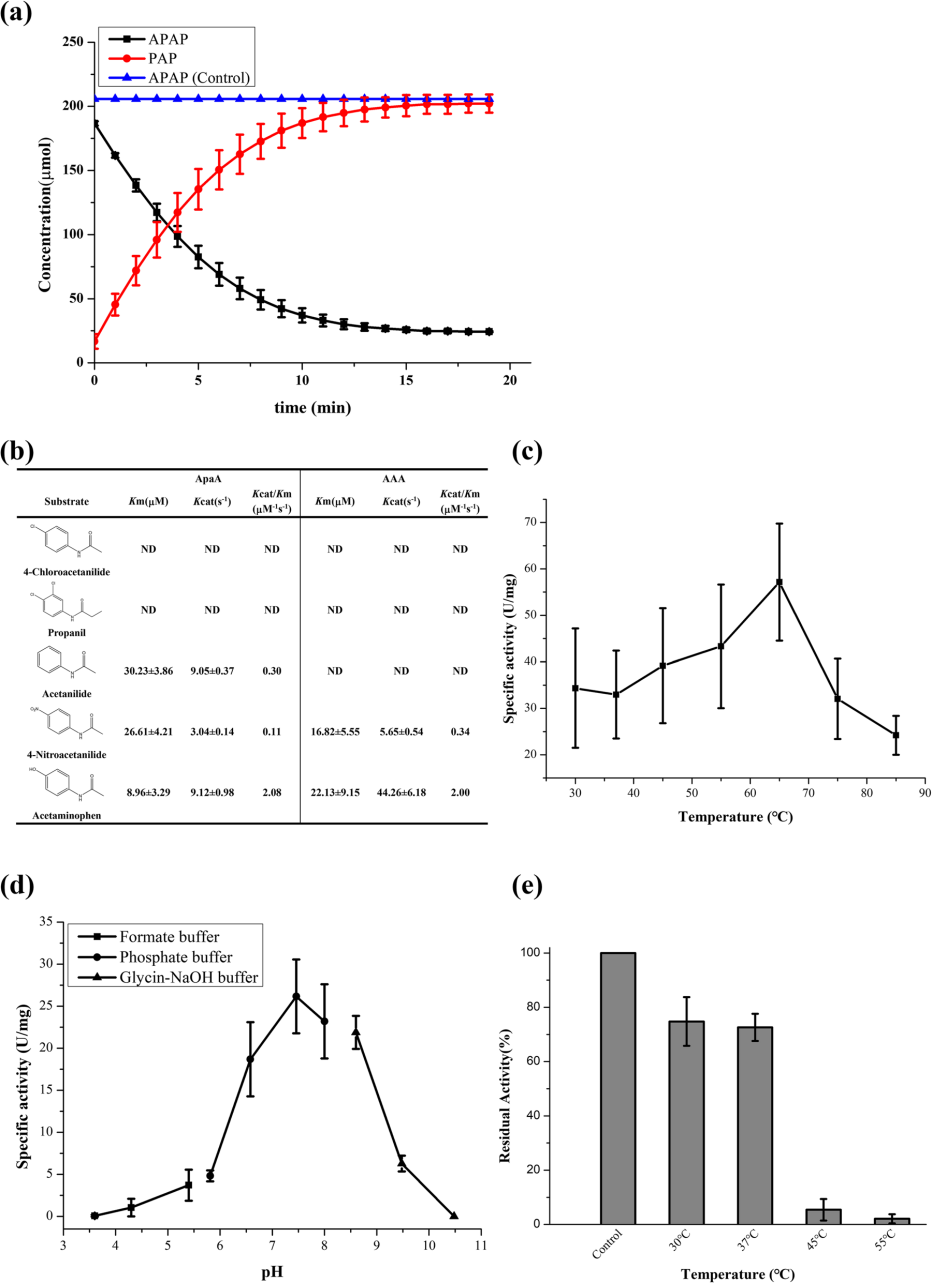
Characterization of the APAP amidase ApaA. **a** Time course of APAP hydrolysis by ApaA. The reaction conditions are detailed in the methods section. **b** Kinetic parameters of amidases AAA and ApaA against various substrates. Temperature-dependence (**c**) and pH-dependence (**d**) activity of ApaA. Enzyme activity was measured at different temperatures and pH levels. Different buffers with different pH ranges were used for enzymatic assay. Error bars represent one standard deviation from the mean of three technical replicates. **e** Thermal stability analysis of ApaA. The activity was assessed after incubating the enzyme at various temperatures for 30 min

The genome sequencing of strain NyZ502 provides valuable insights into its potential mechanisms for APAP degradation. BLASTP analysis revealed a putative amidase designated ApaA associated with APAP hydrolysis, which showed moderate identity (30–36%) with a number of reported APAP amidases (Additional file 1: Table S2 and Additional file 1: Fig. S5). Notably, an amidase (GenBank accession number: K9NBS6.1) from *Rhodococcus erythropolis* TA37 [52] was found to share 97% sequence identity with ApaA. However, it was only known for hydrolyzing various *N*-substituted amides, but whether is active against APAP was not yet confirmed. ApaA from strain NyZ502 was then overexpressed and purified in the homologous host *Rhodococcus erythropolis* L88 [53], and its activity was confirmed by assay using a UV–vis spectrometer (Additional file 1: Fig. S6). Remarkably, during the conversion of APAP to PAP, APAP was completely converted to an approximately stoichiometric equivalent of PAP within 20 min (Fig. 4a). This highlights ApaA as the functional enzyme with evident activity in the APAP degradation. ApaA activity across various pH levels (Fig. 4c) and temperatures (Fig. 4d) were further assessed against APAP. Its optimum pH and temperature were approximately 7.5 and 65 °C, respectively. Additionally, ApaA was stable at temperatures up to 37 °C for at least 30 min, retaining approximately 70% of its activity, but it was sensitive to temperatures above 45 °C (Fig. 4e).

AAA, as a well-studied APAP amidase, has been shown to be closely related to the degradation of APAP at both physiological and biochemical levels [9, 31]. Therefore, it was chosen for comparison of activity with ApaA in this study. Both ApaA and AAA were purified and then subjected to kinetic analysis using various amide-containing substrates as illustrated in Fig. 4b. Neither enzyme was active against propanil (also known as *N*-(3,4-dichlorophenyl)) or 4-chloroacetanilide. ApaA was active towards acetanilide but AAA was not. Given the fact that both enzymes were active against 4-nitroacetanilide and APAP, it can be tentatively concluded that the moiety of *para*-substitution is crucial for their amidase activity. Both ApaA and AAA displayed a similar catalytic efficiency towards APAP, but they achieved this in different ways: ApaA has a higher affinity for APAP (*K*_m_ = 8.96 µM, significantly lower than AAA’s *K*_m_ of 22.13 µM), while AAA’s catalytic efficiency is due to a higher substrate turnover rate (indicated by a notably higher *K*_cat_ compared to ApaA).

### ApaA_alikes are highly abundant in WWTPs

To determine the distribution profile of ApaA in WWTPs, it was searched against sequenced WWTP metagenomes using the same parameters as described earlier. Surprisingly, the homologs of ApaA were found to be highly abundant, with levels even exceeding most of the previously reported functional APAP amidases (Fig. 1b, 1b’). This suggests that ApaA represents an important, yet overlooked, component in APAP degradation in WWTPs. To further assess whether the ApaA characterized here contributes to the diversity and sequence abundance of potential APAP amidases, retrieved homologs of all reported APAP amidases excluding ApaA were compared with those of all reported APAP amidases including newly identified ApaA. These two retrievals were conducted using consistent alignment parameters, forming two separate SSNs (Fig. 2a, a’). The query including ApaA led to the retrieval of 2550 sequences, compared to 1596 sequences when excluding ApaA. Interestingly, the ApaH2-containing cluster was the most abundant in the WWTP metagenomes for both datasets. When the ApaA was included, dramatically increased homologs were found to be clustered with ApaH2. These results highlight the significant contribution of ApaA_alikes to the diversity and sequence abundance of APAP amidases in WWTPs, underscoring its potential role in the functional microbiome of WWTPs.

### Profile of APAP degradation genes in reconstructed MAGs

To gain deeper insights into the genomic context and potential functional variations within specific microbial taxa involved in APAP degradation, we conducted a metagenome-assembled genome (MAG)-level analysis. A total of 211 high-quality MAGs were successfully reconstructed, with over 90% completeness and less than 5% contamination. These MAGs collectively accounted for 0.80 Gbp, with completeness ranging from 90.02 to 100% and contamination levels between 0% and 4.98%. Taxonomic classification assigned all 211 MAGs to the domain Bacteria, spanning 16 bacterial phyla. Detailed characteristics of these MAGs are provided in Additional file 2: Table S4.

**Fig. 5:**
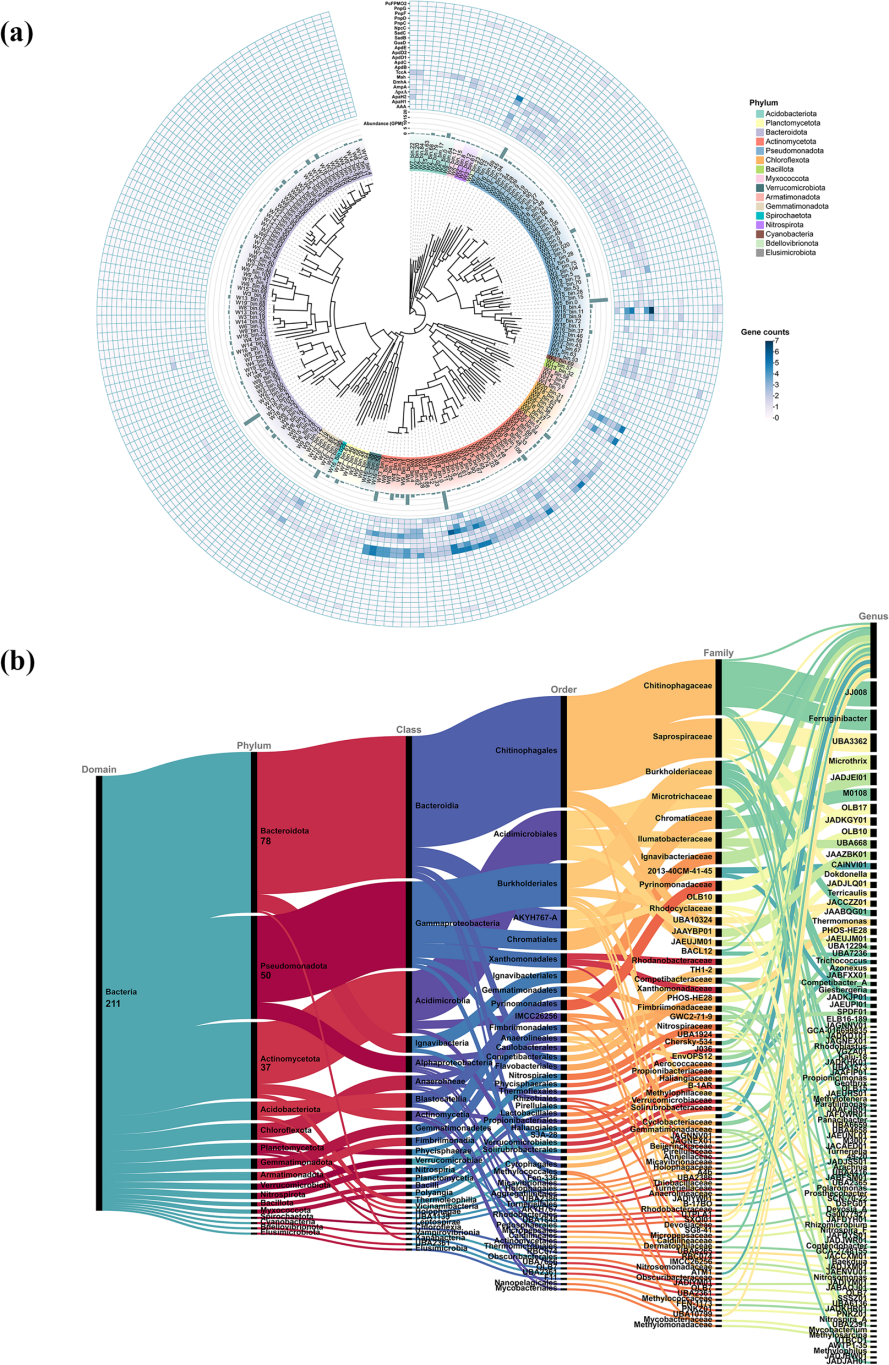
Phylogenetic and functional characterization of high-quality bacterial MAGs reconstructed from WWTP samples. **a** Phylogenetic trees of the reconstructed bacterial MAGs, with different background colors representing phylum-level taxonomic annotations. Abundance values were computed as genome copies per million reads (GPM). Detailed information on the MAG abundance per metagenomic sample can be found in Additional file 2: Table S4. The outer rings indicate the gene counts for each APAP degradation gene in each MAG. **b** A complete domain-to-genus taxonomy for the 211 constructed bacterial MAGs, based on GTDB classification [29]

Among the reconstructed MAGs (Fig. 5b), the phyla Bacteroidota (78 MAGs), Pseudomonadota (50 MAGs), and Actinomycetota (37 MAGs) were the most dominant ones. Other identified phyla include Acidobacteriota (9 MAGs), Chloroflexota (8 MAGs), Planctomycetota (6 MAGs), Gemmatimonadota (5 MAGs), Armatimonadota (4 MAGs), Verrucomicrobiota (3 MAGs), Nitrospirota (3 MAGs), Bacillota (2 MAGs), Myxococcota (2 MAGs), Spirochaetota (1 MAG), Cyanobacteria (1 MAG), Bdellovibrionota (1 MAG), and Elusimicrobiota (1 MAG). The relative abundances of these MAGs varied across the metagenomes of different WWTP samples (Fig. 5a).

Phylogenetic analysis based on the relative abundances of APAP degradation genes within the MAGs revealed corresponding clustering patterns at the phylum level (Fig. 5a). Consistent with metagenome-level findings, a biased distribution of APAP degradation genes in the entire pathway was also observed at the MAG level. Specifically, the APAP amidase genes, including *apaA* characterized in this study, are significantly more abundant than the genes for the downstream degradation. The newly identified *apaA* is predominantly found in the Actinomycetota phylum. This heterogeneity in the composition of the genes for complete APAP degradation within the MAGs suggests that taxonomic distinctions in a way play a critical role in APAP catabolism.

### Sequence and structure diversity of amidases in WWTPs

**Fig. 6:**
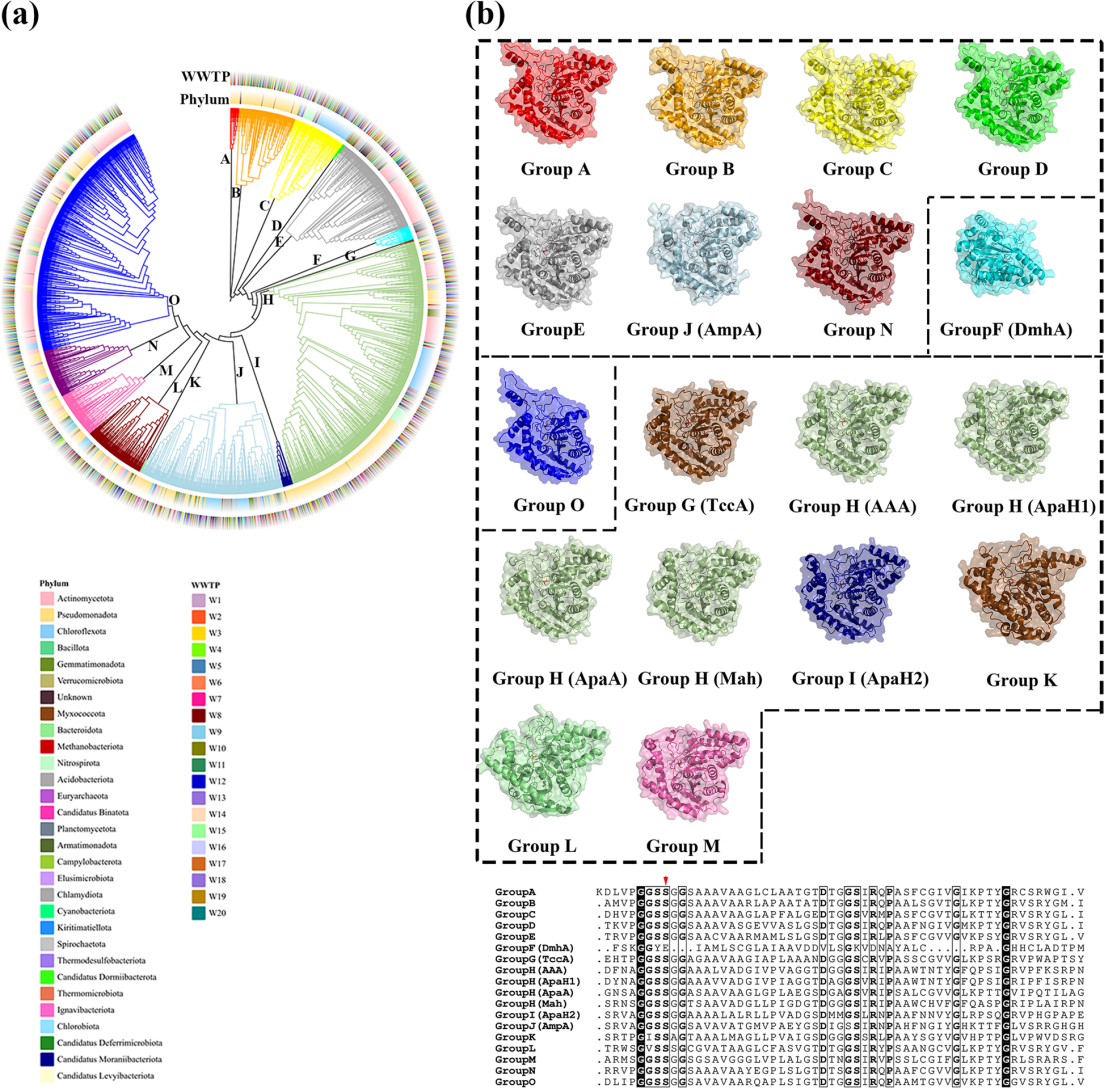
Phylogenetical and structural diversity of putative APAP amidases from WWTPs. **a** Phylogenetic tree of putative APAP amidase candidates (2550 sequences) retrieved from WWTP metagenomes via BLASTP, along with known functional amidases. The outer rings indicate the sampling source and taxonomy of these sequences. Detailed sequence and annotation information are provided in Additional file 3: Table S5. **b** Structural diversity of putative APAP-active amidases and representative enzymes from selected phylogenetic groups. Structural models are shown as cartoons with transparent accessible surface areas, and putative active sites are highlighted with the serine catalytic residue depicted as red sticks. Groups with distinct structural folds are separated by dashed lines. Multiple sequence alignments of the representative enzymes are shown, with conserved residues in the active sites highlighted, and the serine catalytic residue indicated by a red arrow

To further investigate the evolutionary relationships and structural differences among potential APAP amidases in WWTPs, a phylogenetic analysis was conducted based on sequences shown in Fig. 2a’. These sequences, together with 8 known functional APAP amidases, were grouped into distinct phylogenetic clusters (Fig. 6a). The analysis identified at least 15 distinct phylogenetic groups (Groups A to O), with no apparent sample-specific bias, suggesting a consistent pattern of sequence diversity across all WWTP samples. Among these groups, functionally identified amidases were predominantly found in Groups F, G, H, I, and J, including DmhA (Group F) [12], TccA (Group G) [14], ApaH2 (Group I) [15], and AmpA (Group J) [11]. Notably, Group H was the most abundant one, containing sequences of ApaA as well as AAA [9], ApaH1 [15], and Mah [13], suggesting that this group represents a particularly prevalent set of amidases across WWTPs. Amidases were revealed to be extremely abundant in the phylum Actinomycetota, especially those from Groups E, O, and parts of Group H. Sequences in the phylum Pseudomonadota were also widely distributed across multiple groups, including Groups A, B, I, and parts of Group H. These findings indicate that WWTPs of various sources harbor a diverse array of amidases that likely contribute to the adaptive microbial degradation of APAP.

Given the substantial sequence diversity observed (Fig. 6a), the structural diversity within these groups of amidases was further explored by leveraging AlphaFold3 [38] predictions (Fig. 6b). Structural analysis revealed at least four distinct protein folds across the amidase groups. Groups A, B, C, D, E, J (containing AmpA), and N exhibited a highly similar structural fold, while Group G (containing TccA), H, I (containing ApaH2), K, and L shared a different conserved fold, indicating a common core architecture within these groups. Groups O and F (containing DmhA) each exhibited unique folds. Notably, DmhA in Group F with APAP amidase activity did not display a typical catalytic center, further underscoring the structural variation in these enzymes.

These findings highlight the substantial structural diversity among amidases in WWTPs, including large core deletions, modifications, and substantial fold extensions or additions. This structural variability suggests that amidases in WWTPs may exhibit diverse enzymatic functions and substrate specificities, contributing to the complex microbial catabolism of APAP in geographically different WWTPs.

### Global distribution of APAP degradation enzymes in WWTPs

**Fig. 7:**
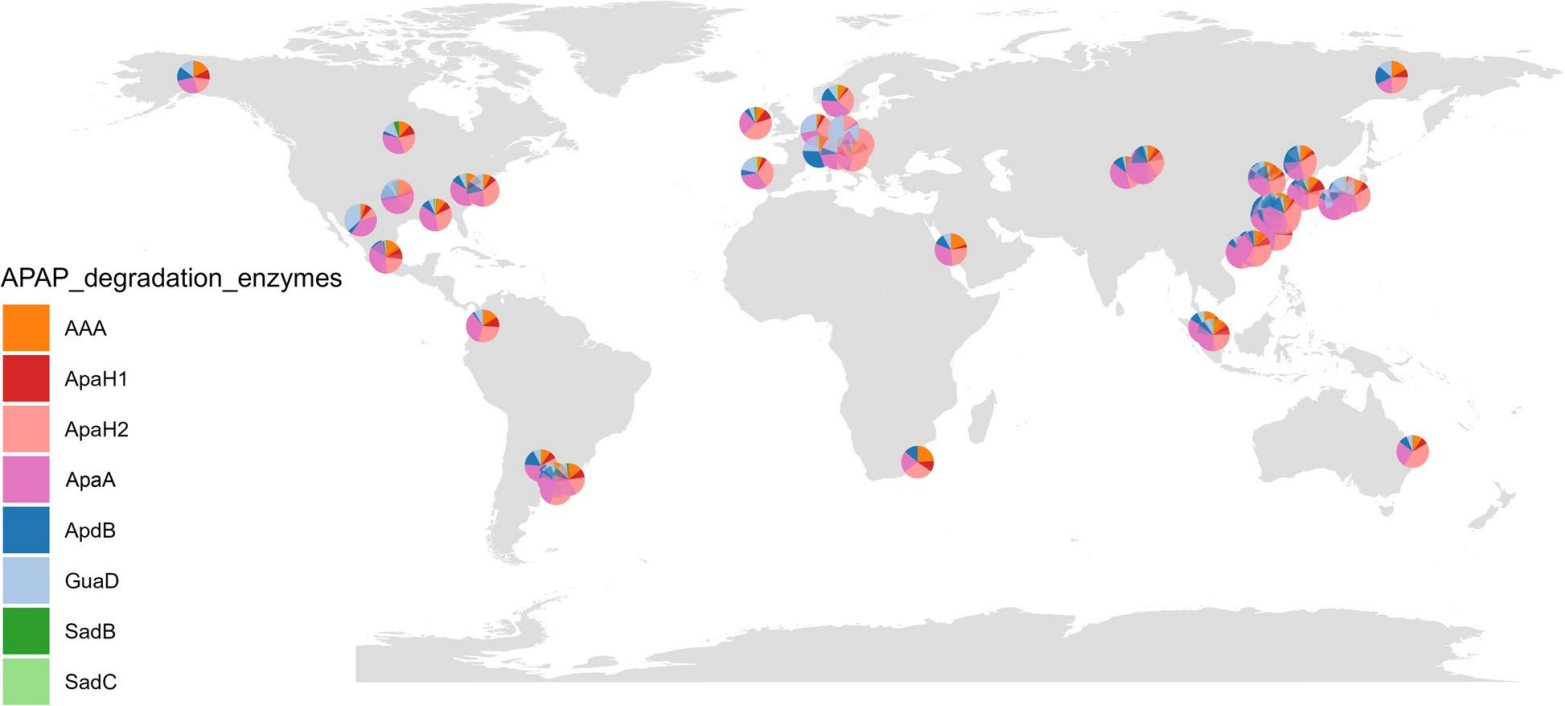
Global distribution of APAP degradation enzymes in available WWTP metagenomes. The distribution of potential APAP degradation enzymes across global WWTP metagenomes is visualized on a world map. For each site, the relative abundance of each enzyme is represented in pie charts. Detailed information is provided in Additional file 4: Table S6

To investigate the global distribution of APAP degradation enzymes across WWTPs, raw sequencing reads from 117 WWTP samples representing diverse geographical locations (Additional file 4: Table S6) were downloaded from the NCBI SRA database and assembled to create a global WWTP metagenome dataset. A BLASTP search was performed against this dataset using enzyme sequences from APAP-utilizing microorganisms involved in APAP degradation. The query sequences include the initial APAP amidases (ApaA, AAA [9], ApaH1, and ApaH2 [15]) and enzymes involved in PAP conversion, including SadBC [40], ApdB [15], and GuaD (an unconfirmed PAP degradation enzyme) [39]. As illustrated in Fig. 7, the initial APAP amidases, particularly ApaA and ApaH2, were found to be significantly more abundant than the downstream enzymes at most sites. These findings indicate a globally uneven distribution of APAP degradation enzymes, echoing that observed in WWTPs sampled across China.

## Discussion

Our study offers novel insights into the environmental degradation of APAP in WWTPs, revealing a diverse array of functional genes involved in this process. The broad distribution of diverse degrading amidases in APAP degradation is a unique characteristic in a complex microbial ecosystem of WWTPs. Additionally, it was also found that the significant accumulation of the intermediate product *p*-aminophenol (PAP) is due to an unanticipated imbalance between genes responsible for upstream and downstream degradation of APAP.

Metagenomic analysis across multiple WWTPs in China uncovered significant diversity in APAP-degrading genes. The predominance of initial degradation enzymes, particularly amidases like ApaA, underscores the widespread and efficient hydrolysis of APAP in these sites [43]. However, the relative scarcity of downstream degradation enzymes, such as those responsible for converting PAP into less harmful compounds, represents a bottleneck in the complete mineralization of APAP. This imbalance in enzyme distribution likely contributes to the observed accumulation of more toxic PAP [41] in the WWTPs. Moreover, certain bacteria in these WWTPs, possessing promiscuous amidases involved in the catabolism of non-APAP *N*-substituted amides (e.g., pesticides) [11, 12], also exhibit activity against APAP, but without functional PAP-degrading enzymes. This may be one of the underlying reasons for the imbalance between the APAP-degrading enzymes and downstream degradation enzymes, ultimately leading to the accumulation of PAP in WWTPs.

Our results corroborate previous studies demonstrating the diverse metabolic pathways of environmental microorganisms for xenobiotic degradation [54]. The widespread presence of amidase genes across various phyla, particularly Actinomycetota and Pseudomonadota, suggests a high potential for horizontal gene transfer (HGT) [55] among microbial communities in multi-source converged reservoirs such as WWTPs. This transfer is likely driven by selective pressure from pharmaceutical pollutants such as APAP. Notably, the presence of identical *aaa* genes, exhibiting 100% nucleotide identity, in Gram-negative genera such as *Comamonas* [10], *Pseudomonas* [9], and *Pandoraea* of this study, strongly suggests the occurrence of HGT events within these Pseudomonadota phyla. On the other hand, due to the genetic barrier between Gram-positive and Gram-negative strains [56], the APAP degradation genes predominantly found in Gram-positive strains such as *apaA* are not present in Gram-negative strains where other types of amidases such as AAA are dominant.

The accumulation of PAP in WWTPs raises significant environmental and public health concerns, because PAP is more toxic than its parent compound APAP, with known hepatotoxic and potentially mutagenic effects [41]. Its persistence in the environment could lead to increased ecological risks, particularly through bioaccumulation in aquatic organisms and subsequent entry into the human food chain. The high concentrations of PAP detected in our samples underline the need for improved treatment processes in WWTPs to ensure the complete degradation of APAP and its byproducts.

The identification of functional genes and their uneven distribution patterns offers decisive targets for developing enhanced bioremediation strategies of APAP polluted sites. For instance, engineered microbial consortia [57] with flux-optimized expression of initial and downstream APAP-degrading enzymes could achieve the breakdown of this pharmaceutical, reducing the accumulation of harmful intermediates PAP. Such engineered microbial consortia could be developed to express an optimized balance of initial APAP-degrading enzymes (e.g., amidases like ApaA) and downstream enzymes (e.g., deaminases targeting PAP). By employing synthetic biology techniques, microbial strains could be designed to overexpress PAP-degrading enzymes, improving the conversion rate of PAP into less-toxic compounds. Additionally, flux optimization [58] will also ensure the coordinated expression of these enzymes to enhance the overall degradation efficiency of APAP. Moreover, the discovery of the enzyme ApaA, with its distinct substrate specificity and activity profile, presents opportunities for biotechnological applications, including the design of more effective catalysts for APAP degradation.

In addition to engineered consortia, bioreactor-based approaches can be employed to optimize biodegradation conditions (e.g., pH, temperature, and nutrient concentration) to favor both the hydrolysis of APAP and the downstream conversion of PAP. A continuous-flow reactor system, for instance, can maintain optimal conditions for microbial growth and enzyme activity, while adaptive laboratory evolution [59] can be also employed to continuously enhance microbial strains for better PAP degradation efficiency. Furthermore, integrating metabolic pathway modeling [60] and microbiome modeling [61] can guide the selection of optimal strains and biodegradation settings to maximize the breakdown efficacy of both APAP and PAP.

Although the development of engineered microbial consortia and bioreactor-based solutions holds great promise, several challenges still remain. For instance, the scalability of these systems in large WWTPs requires careful consideration of microbial stability and the integration of new technologies with existing infrastructures. Additionally, cost-effectiveness will be a critical factor in determining the practical deployment of these bioremediation strategies.

## Conclusions

This study sheds light on the complex dynamics of APAP degradation in wastewater treatment plants (WWTPs), emphasizing the diverse microbial pathways that facilitate this process. The discovery of a wide array of APAP-degrading genes, including the distinct amidase ApaA, underscores the crucial role of microbial communities in initiating the breakdown of APAP. However, the accumulation of PAP, a more toxic intermediate, reveals a bottleneck in the complete degradation pathway. These findings highlight the urgent need for enhanced treatment strategies to address pharmaceutical pollutants and mitigate their environmental and public health impacts. The identification of specific enzymes and their diverse activities presents promising opportunities for developing advanced bioremediation technologies to manage APAP and its byproducts more efficiently in wastewater.

## Supplementary Information

Additional file 1: Figure S1 Calibration curve of APAP and PAP concentrations. Figure S2. Isolation of APAP degrading bacterial strains. Bacterial strains were cultured in APAP-containing basal media. Figure S3. An agarose gel showing PCR products from amplification of reported *aaa* gene in the isolated bacterial strains. Figure S4. Mass spectra of APAP and its degradation intermediate PAP. Figure S5. Phylogenetic analysis of functional APAP amidases. Figure S6. APAP amidase activity assay of ApaA by UV-vis spectrometer. Absorbance change from 257 nm (λmax of APAP) to 229 nm (λmax1 of PAP) and absorbance increase at 294nm (λmax2 of PAP) are shown. Table S1 Concentrations of APAP and PAP at each WWTP site Table S2 BLASTP analysis of ApaA with reported APAP amidases Table S3 Bacterial strains, plasmids and primers used in this study

Additional file 2: Table S4. Detailed characteristics of the constructed MAGs

Additional file 3: Table S5. Detailed sequence and annotation information used in the phylogenetic tree

Additional file 4: Table S6. Abundance of each APAP degradation enzyme in 117 WWTP samples from diverse geographical locations

## Data Availability

The genome of *Rhodococcus* sp. strain NyZ502 has been deposited in the NCBI GenBank under BioProject accession number PRJNA1147891, and the accession number of amidase ApaA is WP_372024545.1. Additionally, the metagenomic data have been deposited in NCBI under BioProject accession number PRJNA1148826.

## Abbreviations

APAP: Acetaminophen
EFI-EST: Enzyme Function Initiative Enzyme Similarity Tool
ESI: Electrospray ionization
GPM: Genome copies per million reads
HGT: Horizontal gene transfer
HPLC: High-performance liquid chromatography
LC-MS: Liquid chromatography-mass spectrometry
MAGs: Metagenome-assembled genomes
MM: Mineral salts medium
MRM: Multiple reaction monitoring
NAPQI: *N*-Acetyl-*p*-benzoquinone imine
PAP: *p*-Aminophenol
QqQ: Triple quadrupole
SDS-PAGE: Sodium dodecyl sulfate–polyacrylamide gel electrophoresis
SRA: Short Read Archive
SSN: Sequence similarity network
TCC: Triclocarban
TOF-MS: Time-of-flight mass spectrometer
TPM: Transcripts per kilobase of exon model per million mapped reads
WWTPs: Wastewater treatment plants
