# Supplementary material for: The universal accumulation of p-aminophenol during the microbial degradation of analgesic and antipyretic acetaminophen in WWTPs: a novel metagenomic perspective
Source: Microbiome. 2025 Mar 7;13:68. doi: 10.1186/s40168-025-02065-2 (PMC11887370; doi:10.1186/s40168-025-02065-2)
Supplement: Supplementary file 2 — Additional file 1: Figure S1 Calibration curve of APAP and PAP concentrations. Figure S2. Isolation of APAP degrading bacterial strains. Bacterial strains were cultured in APAP-containing basal media. Figure S3. An agarose gel showing PCR products from amplification of reported aaa gene in the isolated bacterial strains. Figure S4. Mass spectra of APAP and its degradation intermediate PAP. Figure S5. Phylogenetic analysis of functional APAP amidases. Figure S6. APAP amidase activity assay of ApaA by UV-vis spectrometer. Absorbance change from 257 nm (λmax of APAP) to 229 nm (λmax1 of PAP) and absorbance increase at 294nm (λmax2 of PAP) are shown. Table S1 Concentrations of APAP and PAP at each WWTP site Table S2 BLASTP analysis of ApaA with reported APAP amidases Table S3 Bacterial strains, plasmids and primers used in this study [file 40168_2025_2065_MOESM1_ESM.docx]

**Supplementary information**

**The universal accumulation of *p*-aminophenol during the microbial degradation of** **analgesic and antipyretic acetaminophen in WWTPs: A novel metagenomic perspective**

Chao-Fan Yin^1#^, Piaopiao Pan^1#^, Tao Li^1^, Xin Song^2^, Ying Xu^1*^, Ning-Yi Zhou^1*^

^1^State Key Laboratory of Microbial Metabolism, Joint International Research Laboratory of Metabolic & Developmental Sciences, and School of Life Sciences & Biotechnology, Shanghai Jiao Tong University, Shanghai, 200240, China.

^2^State Key Laboratory of Soil and Sustainable Agriculture, Institute of Soil Science, Chinese Academy of Sciences, Nanjing, 211135, China

^*^Corresponding author: Ying Xu (ying.xu@sjtu.edu.cn) and Ning-Yi Zhou (ningyi.zhou@sjtu.edu.cn).

Mailing address: 800 Dongchuan Road Shanghai, Shanghai Jiao Tong University, China, 200240. Tel.: +86-21-34204135. Fax: +86-21-34208261.+

#These authors contribute equally to this work.


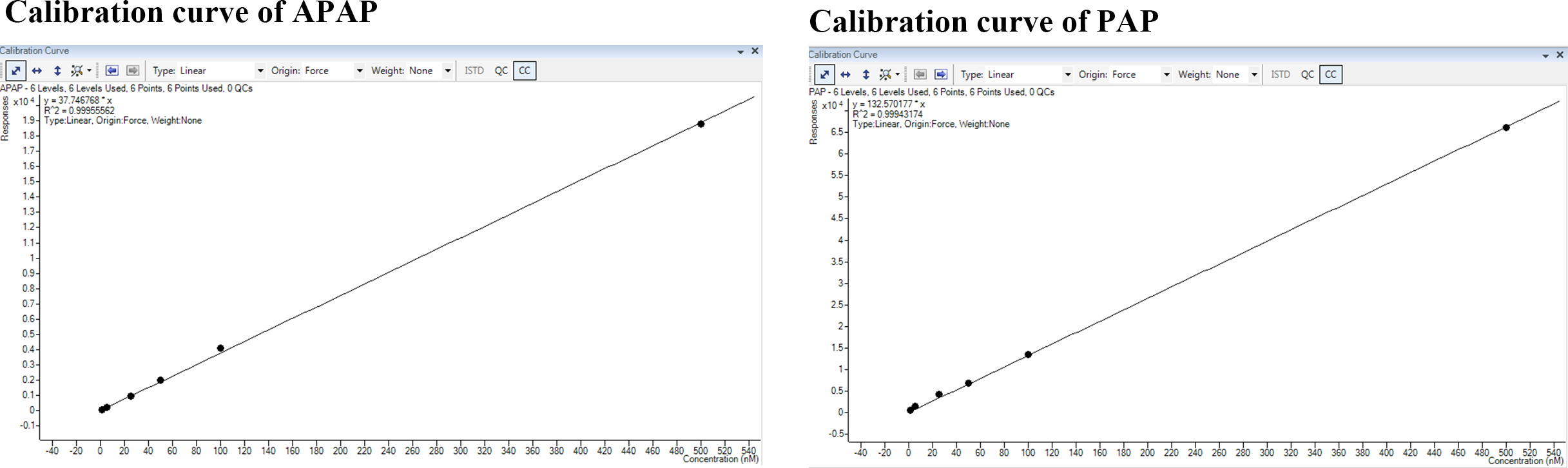


**Fig. S1 Calibration curve of APAP and PAP concentrations**


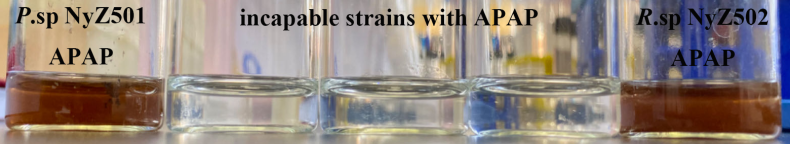


**Fig. S2 Isolation of APAP degrading bacterial strains.** Bacterial strains were cultured in APAP-containing basal media.


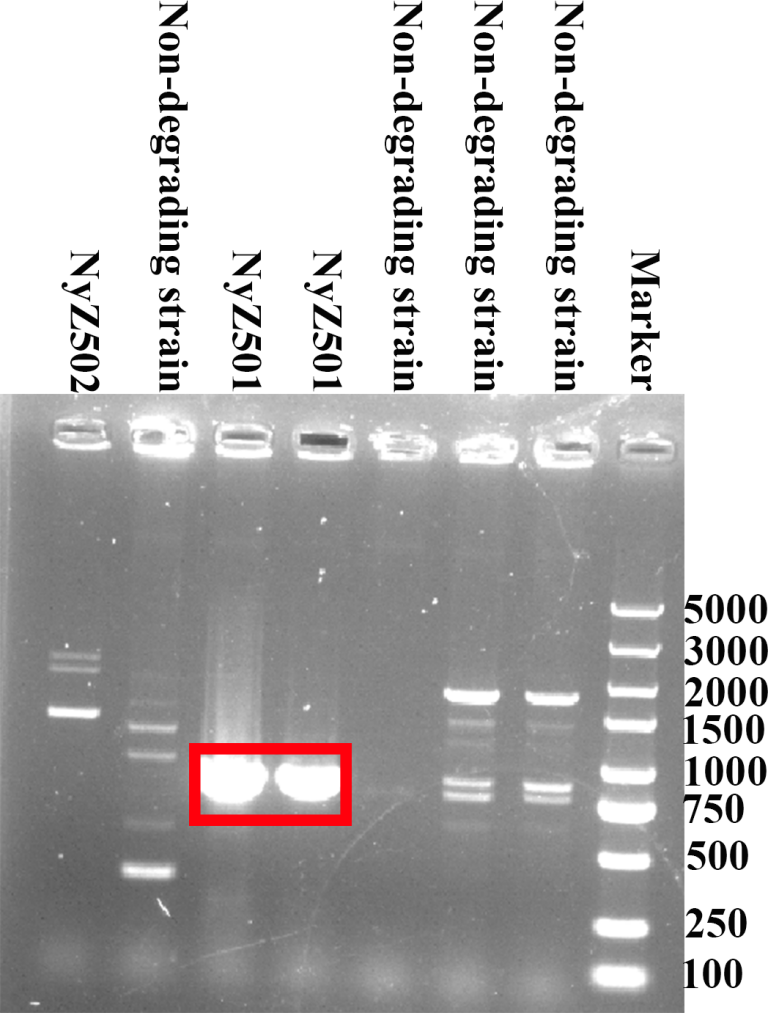


**Fig. S3 An agarose gel showing PCR products from amplification of reported *aaa* gene in the isolated bacterial strains.**

**
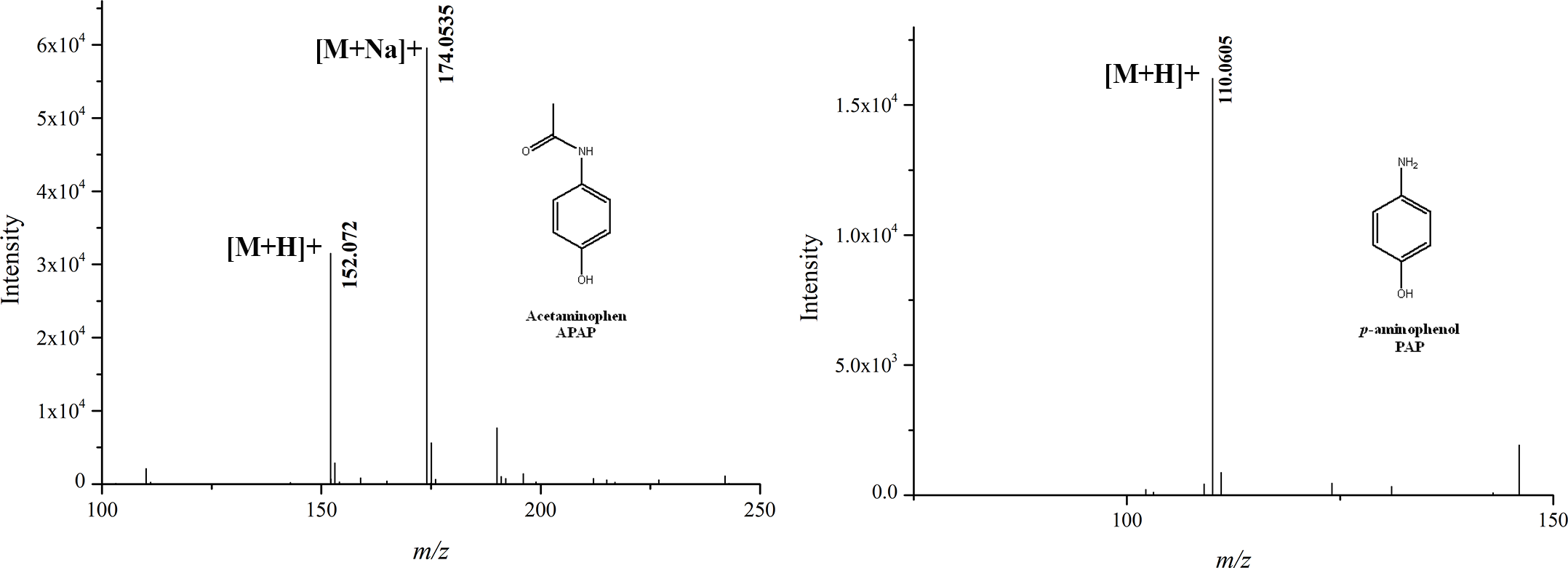
**

**Fig. S4 Mass spectra of APAP and its degradation intermediate PAP.**


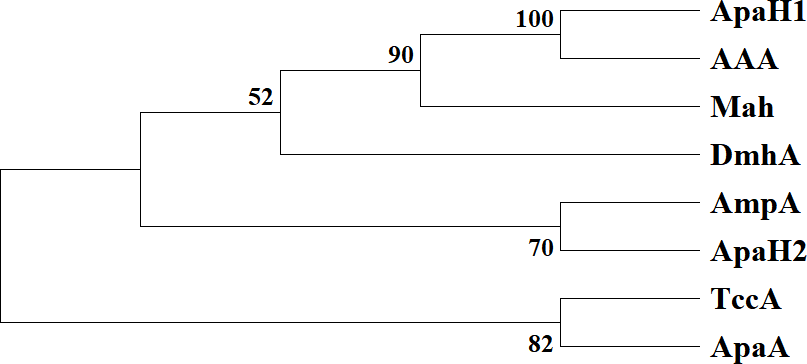


**Fig. S5 Phylogenetic analysis of functional APAP amidases.**


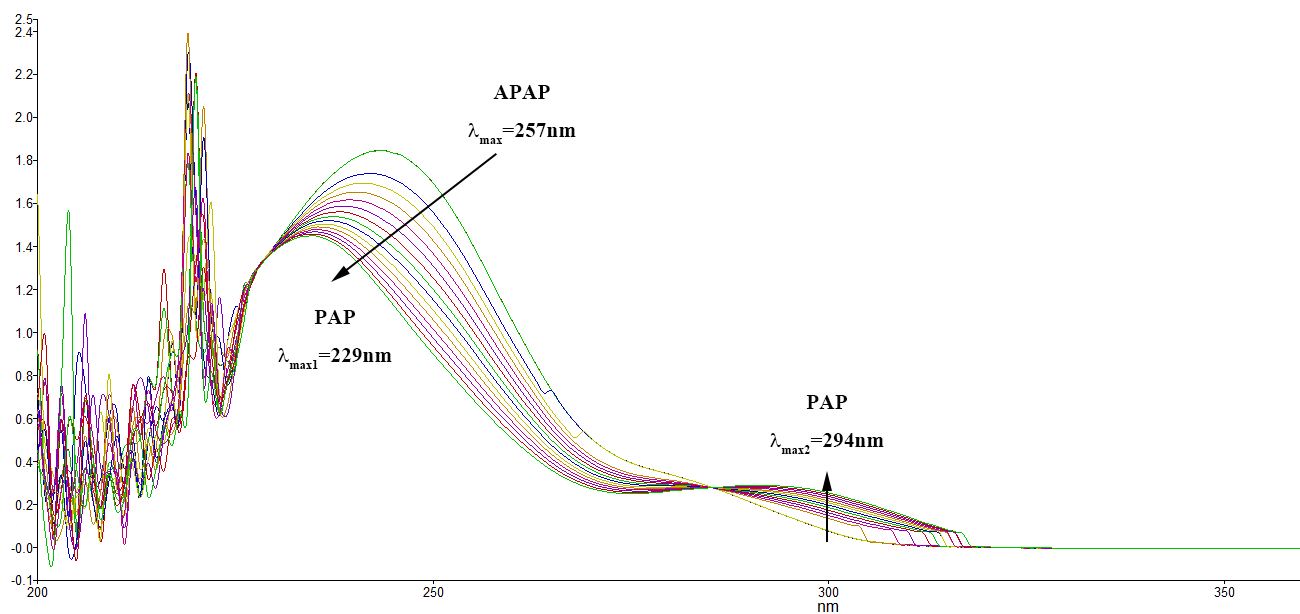


**Fig. S6 APAP amidase activity assay of ApaA by UV-vis spectrometer.** Absorbance change from 257 nm (λ_max_ of APAP) to 229 nm (λ_max1_ of PAP) and absorbance increase at 294nm (λ_max2_ of PAP) are shown.

**Table S1 Concentrations of APAP and PAP at each WWTP site**

| **Num. of sample** | **PAP(nM)** | | **APAP(nM)** | | **T value** | **degree of freedom** | **P value** | **Longitude** | **Latitude** |
| --- | --- | --- | --- | --- | --- | --- | --- | --- | --- |
|  | Average value | Standard deviation | Average value | Standard deviation |  |  |  |  |  |
| W1 | 36.58 | 11.49 | 8.64 | 7.16 | 3.575 | 4 | 0.023 | 108.3807 | 22.83438 |
| W2 | 23.93 | 0.40 | 0.54 | 0.94 | 39.685 | 4 | 0.000002 | 111.3279 | 23.53221 |
| W3 | 108.68 | 9.40 | 16.01 | 12.60 | 10.212 | 4 | 0.001 | 119.968 | 30.27724 |
| W4 | 47.34 | 0.39 | 2.20 | 0.87 | 82.053 | 4 | 1.32E-07 | 120.7002 | 27.72475 |
| W5 | 73.88 | 5.78 | 3.08 | 5.13 | 15.857 | 4 | 0.000092 | 116.9605 | 36.69986 |
| W6 | 49.71 | 2.64 | 2.47 | 1.45 | 27.160 | 4 | 0.000011 | 118.5106 | 32.11239 |
| W7 | 41.79 | 1.14 | 0.06 | 0.11 | 62.678 | 4 | 3.88E-07 | 120.7227 | 31.49377 |
| W8 | 52.61 | 4.91 | 6.00 | 3.78 | 13.037 | 4 | 0.0002 | 121.3394 | 31.33557 |
| W9 | 48.35 | 1.00 | 0.00 | 0 | 83.759 | 2 | 0.000143 | 117.2334 | 31.8256 |
| W10 | 83.59 | 5.67 | 9.49 | 4.65 | 17.489 | 4 | 0.000063 | 103.778 | 36.03972 |
| W11 | 92.28 | 13.31 | 18.14 | 19.47 | 5.445 | 4 | 0.006 | 126.593 | 45.9319 |
| W12 | 68.92 | 7.59 | 0.91 | 0.79 | 15.429 | 4 | 0.000103 | 120.1419 | 31.65188 |
| W13 | 47.88 | 0.89 | 29.20 | 15.78 | 2.048 | 4 | 0.11 | 122.0189 | 39.69178 |
| W14 | 57.06 | 1.02 | 5.57 | 2.80 | 29.907 | 4 | 0.000007 | 119.9976 | 28.88036 |
| W15 | 88.06 | 13.28 | 0.38 | 0.34 | 11.431 | 2 | 0.008 | 113.3565 | 23.05244 |
| W16 | 72.00 | 3.13 | 2.72 | 0.63 | 37.610 | 4 | 0.000003 | 111.2849 | 23.48328 |
| W17 | 77.05 | 7.78 | 4.12 | 4.16 | 14.310 | 4 | 0.000139 | 114.2419 | 30.45569 |
| W18 | 49.28 | 3.05 | 0.52 | 0.91 | 26.510 | 4 | 0.000012 | 106.5584 | 29.56849 |
| W19 | 66.52 | 0.80 | 0.23 | 0.40 | 128.525 | 4 | 2.20E-08 | 125.1092 | 46.59284 |
| W20 | 62.25 | 1.15 | 3.01 | 0.15 | 88.737 | 2 | 0.000099 | 108.3746 | 22.82261 |

**Table S2 BLASTP analysis of ApaA with reported APAP amidases**

| **Amidases** | **Identity with ApaA** | **Evalue** | **Coverage** | **Reference** |
| --- | --- | --- | --- | --- |
| AAA | 33.61% | 5E-67 | 96% | [1] |
| ApaH1 | 33.00% | 9E-67 | 97% | [2] |
| ApaH2 | 31.72% | 7E-54 | 97% | [2] |
| AmpA | 29.91% | 5E-39 | 90% | [3] |
| DmhA | ND | ND | ND | [4] |
| Mah | 35.89% | 1E-77 | 98% | [5] |
| TccA | 35.31% | 6E-62 | 97% | [6] |

ND: not detectable

**Table S3 Bacterial strains, plasmids and primers used in this study**

| **Bacterial strains, plasmids or primers** | **Descriptions or sequences (5’ to 3’)** | **Sources or references** |
| --- | --- | --- |
| **Bacterial strains** |  |  |
| *E.coli* DH5a | lacU169ΔlacU169 (φ80dlacZΔM15) hsdR17 recA1 endA1 hsdR17 thi^-1^ gyrA96 relA1 | Novagen |
| *E. coli* BL21 (DE3) | F^-^ ompT hsdS_B_ (Rb^-^mB^-^) gal (λcI857 ind1 Sam7 nin5 lacUV5-T7gene1) dcm (DE3) | Novagen |
| *Rhodococcus erythropolis* L88 | Expression host of recombinant proteins | [7] |
| *Pandoraea* sp. NyZ501 | Acetaminophen degrader, G^-^ | This study |
| *Rhodococcus* sp. NyZ502 | Acetaminophen degrader, G^+^ | This study |
| **Plasmids** |  |  |
| pET-28a (+) | Overexpression vector; Km^r^ | Novagen |
| pTIPQC1 | Overexpression vector; Cm^r^ | [8] |
| pET-*aaa* | Km^r^; pET-28a derivative for the overexpression of *aaa* | This study |
| pTIP-*apaA* | Cm^r^; pTIPQC1 derivative for the overexpression of *apaA* | This study |
| **Primers^a^** |  |  |
| F(*aaa*) | cgcgcggcagccatATGGGTAAGTCACATTCGCCAG | Forward primer of *aaa* for expression |
| R(*aaa*) | gctcgaattcggatccTCACAGGGGCCGTCCG | Reverse primer of *aaa* for expression |
| F(*apaA*) | atcaccatcaccatatgATGACCGAACAGAATCTGCATTGG | Forward primer of *apaA* for expression |
| R(*apaA*) | atctaagcttggatccCTAGGCCACGACCAGTGAAT | Reverse primer of *apaA* for expression |
| 27F | AGAGTTTGATCCTGGCTCAG | Universal primer for amplification of bacterial full-length 16S rRNA gene |
| 1492R | GGYTACCTTGTTACGACTT | Universal primer for amplification of bacterial full-length 16S rRNA gene |

^a^ Lowercase letters in the primers indicate overlapped sequences of vector.

References:

1. Ko HJ, Lee EW, Bang WG, Lee CK, Kim KH, Choi IG: **Molecular characterization of a novel bacterial aryl acylamidase belonging to the amidase signature enzyme family**. *Mol Cells* 2010, **29**(5):485-492.

2. Zhang M, Zhu W, Liu H, Pan K, Li Q, Zhu Q, Huang Y, Wang C, Hu J, Jiang M *et al*: **Unveiling the analgesic and antipyretic drug acetaminophen catabolic mechanism in Pseudomonas taiwanensis AP-1**. *J Hazard Mater* 2024, **480**:136281.

3. Zhang J, Yin JG, Hang BJ, Cai S, He J, Zhou SG, Li SP: **Cloning of a novel arylamidase gene from Paracoccus sp. strain FLN-7 that hydrolyzes amide pesticides**. *Appl Environ Microbiol* 2012, **78**(14):4848-4855.

4. Chen Q, Chen K, Ni H, Zhuang W, Wang H, Zhu J, He Q, He J: **A novel amidohydrolase (DmhA) from Sphingomonas sp. that can hydrolyze the organophosphorus pesticide dimethoate to dimethoate carboxylic acid and methylamine**. *Biotechnol Lett* 2016, **38**(4):703-710.

5. Zhang L, Hu Q, Hang P, Zhou X, Jiang J: **Characterization of an arylamidase from a newly isolated propanil-transforming strain of Ochrobactrum sp. PP-2**. *Ecotoxicol Environ Saf* 2019, **167**:122-129.

6. Yun H, Liang B, Qiu J, Zhang L, Zhao Y, Jiang J, Wang A: **Functional Characterization of a Novel Amidase Involved in Biotransformation of Triclocarban and its Dehalogenated Congeners in Ochrobactrum sp. TCC-2**. *Environ Sci Technol* 2017, **51**(1):291-300.

7. Yoshida K, Kitagawa W, Ishiya K, Mitani Y, Nakashima N, Aburatani S, Tamura T: **Genome Sequence of Rhodococcus erythropolis Type Strain JCM 3201**. *Microbiol Resour Announc* 2019, **8**(14).

8. Nakashima N, Tamura T: **A novel system for expressing recombinant proteins over a wide temperature range from 4 to 35°C**. *Biotechnology and Bioengineering* 2004, **86**(2):136-148.
